# Construction of edit-distance graphs for large sets of short reads through minimizer-bucketing

**DOI:** 10.1093/bioadv/vbaf081

**Published:** 2025-04-10

**Authors:** Pengyao Ping, Jinyan Li

**Affiliations:** School of Computer Science, Faculty of Engineering and Information Technology, University of Technology Sydney, Ultimo, NSW 2007, Australia; School of Computer Science, Faculty of Engineering and Information Technology, University of Technology Sydney, Ultimo, NSW 2007, Australia; School of Computer Science and Control Engineering, Shenzhen University of Advanced Technology, Shenzhen, Guangdong 518000, China

## Abstract

**Motivation:**

Pairs of short reads with small edit distances, along with their unique molecular identifier tags, have been exploited to correct sequencing errors in both reads and tags. However, brute-force identification of these pairs is impractical for large datasets containing ten million or more reads due to its quadratic complexity. Minimizer-bucketing and locality-sensitive hashing have been used to partition read sets into buckets of similar reads, allowing edit-distance calculations only within each bucket. However, challenges like minimizing missing pairs, optimizing bucketing parameters, and exploring combination bucketing to improve pair detection remain.

**Results:**

We define an edit-distance graph for a set of short reads, where nodes represent reads, and edges connect reads with small edit distances, and present a heuristic method, reads2graph, for high completeness of edge detection. Reads2graph uses three techniques: minimizer-bucketing, an improved Order-Min-Hash technique to divide large bins, and a novel graph neighbourhood multi-hop traversal within large bins to detect more edges. We then establish optimal bucketing settings to maximize ground truth edge coverage per bin. Extensive testing demonstrates that read2graph can achieve 97%–100% completeness in most cases, outperforming brute-force identification in speed while providing a superior speed-completeness balance compared to using a single bucketing method like Miniception or Order-Min-Hash.

**Availability and implementation:**

reads2graph is publicly available at https://github.com/JappyPing/reads2graph.

## 1 Introduction

An edit-distance graph for a set of strings is a graph whose every node represents a string and every edge represents the edit distance of the two nodes less than or equal to a threshold. Efficient construction of an edit-distance graph from a set of short reads or a set of Unique Molecular Identifiers (UMIs) is useful in many applications such as removal or correction of sequencing errors and PCR (Polymerase Chain Reaction) errors contained in the reads or UMIs (Smith *et al.* 2017, Ma *et al.* 2021, Ping *et al.* 2024). The leverage of the edit-distance graph is significant and right for this Next Generation Sequencing (NGS) data noise removal because the PCR amplification and fluorophore crosstalk have resulted in millions of singletons or low-frequency reads that exhibit just minor deviations from their true state (Kebschull and Zador 2015, Ma *et al.* 2019). The edit distance [Levenshtein distance (Levenshtein 1966)] happens to quantify the minimum number of single-base edits required to transform one sequence into another, just encompassing the base insertions, deletions, and substitutions in the PCR erring and fluorophore crosstalk (Bose *et al.* 2015, Kebschull and Zador 2015). Various methods based on edit distance or Hamming distance (a special case of edit distance) have been previously developed to eliminate duplicated reads or to correct PCR and sequencing errors. For example, the deduplication method named UMI-tools constructs an edit-distance graph for a set of UMI strings to mitigate sequencing errors in these UMI tag sequences (Smith *et al.* 2017). Studies by (Fu *et al.* 2018, Parekh *et al.* 2018, Pflug and von Haeseler 2018, Sun *et al.* 2024) used UMI-tools directly or improved the graph construction strategy in UMI-tools to remove PCR duplicates or rectify PCR amplification errors in UMIs. As another example, our previous method named noise2read finds the 1 nt- or 2 nt-edit-distance edges between unique high-frequency reads and all the other unique reads to construct a graph for correcting erroneous reads (Ping *et al.* 2024).

The time complexity of these methods is very high. UMI-tools conducts exhaustive pairwise comparisons of the UMI strings to build an edit-distance graph (Liu 2019); the method UMIc (Tsagiopoulou *et al.* 2021) conducts pairwise comparisons between high-frequency reads/UMIs and the others for the calculation of UMI-to-UMI distances and read-to-read distances; while noise2read enumerates all possible strings which have a 1 nt- and 2 nt-edit-distance away from every high-frequency read in the graph construction (Ping *et al.* 2024). Although conducting all-vs-all sequence comparisons can yield complete and precise edit-distance graphs, these squared- or exponential-complexity algorithms require substantial computing resources and are prohibitively impractical for handling large-scale sets of short reads or when the reads have longer lengths.

In this work, we introduce a heuristic and efficient method with various minimizer bucketing modes to build an edit-distance graph from a large-scale set R of reads or a set M of UMIs. As it is a heuristic method, some of the edges may not be detected for the graph. However, the proposed algorithm can run and finish the construction of edit-distance graphs with superior balance between completeness and running time. The key step of the heuristic method is to divide R into nonoverlapping subgroups $R_{\mathrm{sub}\_1},R_{\mathrm{sub}\_2},\ldots$ such that the reads’ similarity within each subgroup is very high. Then, we compute the edit distance for every pair of reads within each subgroup $R_{\mathrm{sub}\_i}$. Thus, the complexity of determining the edges of the graph can be reduced from $O(|R{|}^{2})$ to $O(|R_{\mathrm{sub}\_i}{|}^{2})$. In detail, we use random minimizer and Miniception (Zheng *et al.* 2020) with or without read segmentation to bucket all the reads in R into subgroups. In case when some of these subgroups still contain a large amount of reads, we improve an Order-Min-Hash (OMH) (Marçais *et al.* 2019) string allocation technique to divide such big subgroups. In rare cases of still having big subgroups of reads that cannot be divided into normal-sized bins even after the two-layer bucketing, new edges from such ‘rigid’ big subgroups are generated by a novel graph traversal technique that implements a ‘triangle inequality’ distance principle.

Minimizers and Locality-Sensitive Hashing (LSH) have been widely investigated for estimating sequence similarity. Although the minimizer strategy was initially proposed to reduce memory consumption and accelerate sequence overlapping detection (Roberts *et al.* 2004a), it has been shown useful for indexing sequences to facilitate rapid search for matched sequences in read mappers (Ren and Chaisson 2021, Jain *et al.* 2022). LSH has been used to group similar barcodes and reads if their barcodes differ by at most e Hamming differential bases from each other (Orabi *et al.* 2019). The study by (Liu *et al.* 2021) proposed to generate multiple rounds of clusters of reads in R using minimizers for removing duplicate reads. Some other research has focused on the theoretical design of novel minimizers and LSH schemes. For example, OMH which provides theoretical guarantees as a gapped LSH was proposed to approximate Jaccard and edit distance similarity (Marçais *et al.* 2019). Another study (Chen and Shao 2023) introduced (*d1*, *d2*) -sensitive bucketing functions for the edit distance, where *d1* and *d2* are two thresholds (*d1 *<* d2*), and presented theoretical proofs for various settings of (*d1*, *d2*) for hashing a sequence into multiple buckets. Giulio *et al.* devised a locality-preserving minimal hash function to reduce space usage for hashing consecutive *k*-mers from a collection of sequences (Pibiri *et al.* 2023). The latest advancements in the theories and applications of minimizer-bucketing can be found in a recent survey (Zheng *et al.* 2023).

Specifically in this work, we address the following questions about efficient edge detection from large-scale sets of short reads for building edit-distance graphs:

- We investigate whether the probability of edge detection after minimizer bucketing can be improved using a multi-minimizer-one-read approach, which divides each read into multiple independent parts, compared to the traditional one-minimizer-one-read approach, where minimizers are sampled over the entire read. Additionally, we explore how to optimize the minimizer length and window size for both strategies to maximize edge detection probability. In other words, we aim to achieve the optimal trade-off between speed and completeness in edge detection for a set of short reads by fine-tuning these parameters for different bucketing approaches.
- As sometimes there still exist large buckets holding prohibitive pair-wise computation in practice even after the multi-minimizer-one-read bucketing for R, we investigate whether any other heuristic method differing from minimizer-bucketing is useful to split such large buckets. We introduce a new bucketing step named OMH with gapped k-mers (gOMH) bucketing to further subdivide these large bins. We optimize this bucketing step by a probability analysis on the selection of a gapped k-mer with the minimum hash value to represent a read. We then determine an appropriate k-mer size for each $d_{t}$ in $\left[d_{\min},d_{\max}\right]$ based on this analysis. The gapped k-mers and their ordering can generate different candidates compared to the k-mers used in the minimizer-bucketing, increasing the likelihood of dividing large groups of similar reads into smaller, more manageable clusters.
- To handle large buckets that cannot be divided even by the above two-layer bucketing, we introduce a graph traversal process to make a multi-hop visit on the neighbouring nodes of every unique read in these large buckets to add new edges to the graph through a triangle inequality property. This approach is feasible because some of the reads in these large buckets already have edges generated after the two-layer bucketing. Then multi-hop visits on the neighbouring nodes of these edges are effective to recover some missing edges after checking the edit distances of these edges of these triangles in the visit path.

Given a set R of short reads, we compared the number of edges in its edit-distance graph constructed using the brute-force method, the practical minimizer Miniception (Zheng *et al.* 2020), and the LSH scheme OMH (Marçais *et al.* 2019), with the number of edges in the edit-distance graph constructed by our bucketing and traversal steps. The results show that the number of edges detected by our heuristic method closely approaches the 100% completeness of the ground truth edges while maintaining efficient runtime. The developed method, reads2graph, offers a practical approach for constructing an edit-distance read graph that can be applied to various bioinformatics applications, such as data compression, error correction, and deduplication.

## 2 Methods

### 2.1 Problem formulation

Given an alphabet Σ={A,C,G,T,N} or Σ={A,C,G,U,N}, a short DNA or RNA sequence can be represented as $r=b_{1}b_{2}\ldots b_{l}$, where $b_{i}\in \Sigma$; *A, G, C, T*, and U denote the nitrogenous bases Adenine, Guanine, Cytosine, Thymine, and Uracil, respectively; The symbol N represents an ambiguous nucleotide, and l∈N indicates the length (i.e. total number of bases) of a read r. Then, we use R to denote an NGS dataset consisting of hundreds of thousands of short reads.

The goal of our study is to identify all of those read pairs in a short read set R whose edit distance falls in a specified interval $\left[d_{\min},d_{\max}\right]$, and then use these edges to build an edit-distance graph for R. In most cases, $d_{\min}$ is set as 1, and $d_{\max}$ is set as a small integer number (e.g. 3–5), depending on different application scenarios. In this study, within the context of short-read sequencing data, ‘similar reads’ refer to read pairs originating from the same or nearby genomic loci but differ due to sequencing errors, genetic variants, or mutations. Additionally, terms like ‘differing’, ‘shifting’, ‘mutated’ or ‘unmutated’ describe variations between two similar reads rather than positional shifts within a single read.

### 2.2 Reads2graph’s workflow to build an edit-distance graph from a set R of reads

**Figure 1.**
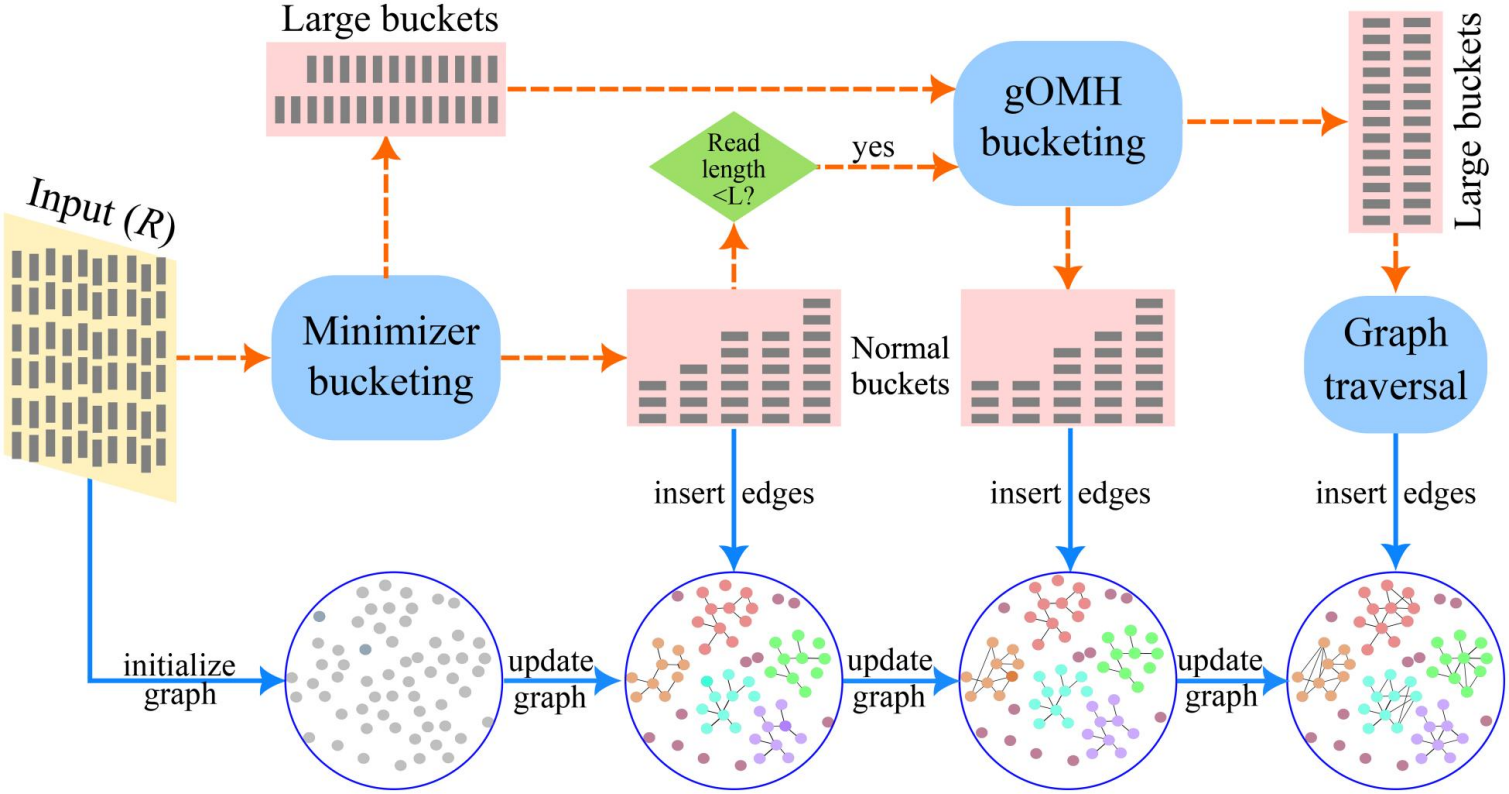
Overview of the workflow of reads2graph. It begins by initializing an undirected graph based on the unique reads from a read set *R.* This initial graph is then progressively updated by inserting new edges. These edges are identified through regular buckets generated via minimizer bucketing, Order Min Hash (gOMH) bucketing with gapped *k*-mers and graph traversal.

We present reads2graph, a heuristic and efficient method for constructing an edit-distance graph for R, which consists of four steps. A flowchart outlining this process is shown in Fig. 1. Specifically, reads2graph initially constructs an undirected graph using the boost C++ (boost v1.82.0) library, where nodes are denoted as numbers with each number corresponding to a unique read, and the sequence itself and its frequency are used as node attributes. The read graph is then updated by the insertion of new edges after the calculation of edit distance between the reads within every subgroup of R as divided by minimizer-bucketing. For those reads that are difficult to subgroup into normal-sized buckets, an OMH with gapped k-mers is used to bucket them to search for additional edges. Finally, if some subgroups of the reads remain very large even after the two layers of bucketing, edges from these reads are detected through a novel graph traversal step.

### 2.3 Minimizer bucketing

A k-mer is a substring from a read $r=b_{1}b_{2}\ldots b_{l}$, consisting of k consecutive nucleotides, where 1≤k≤l. A minimizer-bucketing scheme is characterized by the k-mer length k, a window (substring) size w−k+1 that contains exactly w overlapping k-mers, and a k-mer ordering O. A minimizer is the smallest k-mer in the window under the ordering O.

We incorporate the practical minimizer Miniception (Zheng *et al.* 2020) and the random minimizer into our minimizer bucketing process. Miniception guarantees a density of <2/(w+1) and is applicable for infinitely many values of w and k [see the proof in the original study (Zheng *et al.* 2020)]. The random minimizer is generated by using modules from the efficient C++ library SeqAn3 (version 3.3.0) (Reinert *et al.* 2017, Rahn *et al.* 2018, Hauswedell 2021). This random ordering via hashing will reduce bias toward certain bases, as reported in the literature (Marçais *et al.* 2017). In case there is more than one smallest value within one window, the rightmost one is chosen as the minimizer of the window, and when the window is shifted, the minimizer is only changed if there appears a value that is strictly smaller than the current minimum (Jain *et al.* 2020), which has been proven to work especially well on repeat regions.

A perfect bucketing for R is to subgroup all and only those pairs of reads with an edit distance in the interval [1, $d_{\max}$] into the same bucket. However, minimizer-bucketing cannot provide this perfect guarantee. To optimize the trade-off between speed and completeness in edge detection for a set of short reads, we propose two approaches to minimizer bucketing. The first approach, multi-minimizer-one-read, segments each read into multiple independent parts and applies minimizer sampling to each part separately. The second approach, one-minimizer-one-read, selects k-mers by sliding windows over the entire read.

#### 2.3.1 Bucketing via multi-minimizer-one-read with random minimizer

We assume the $d_{\max}$ bases are evenly distributed between a pair of similar reads. After segmenting a read into n independent parts, each part consists of ⌊l/n⌋ bases. The probability of any k-mer being chosen for bucketing is 1/(⌊l/n⌋−k+1). Given that k−1 bases overlap between consecutive k-mers, a single base shift affects at most k  k-mers within a segment of a read. For two similar reads differing by $d_{\max}$ bases, each independent part of a read contains $d_{\max}/n$ differing bases. Therefore, the probability of selecting an unmutated k-mer from a read is

(1) $$p_{1}\geq \frac{\left\lfloor l/n\right\rfloor -k+1-\frac{d_{\max}}{n}\cdot k}{\left\lfloor l/n\right\rfloor -k+1}.$$

Since each segment’s minimizer generation is independent, the likelihood of multiple minimizers simultaneously containing one of these differing bases is minimal. The probability P of subgrouping two similar reads into the same bucket by at least one minimizer that does not include the differing bases is

(2) $$\begin{array}{c} P(l,k,n,d_{\max})\geq 1-{\left(1-p_{1}\right)}^{n}\\ =1-{\left(\frac{d_{\max}\cdot k}{(\left\lfloor l/n\right\rfloor -k+1)\cdot n}\right)}^{n} \end{array}.$$

For a read set R with read length l, optimal k and w can be determined by maximizing the probability P, formally given by

(3) $$\arg\underset{l,k,n,d_{\max}}{\max}P(l,k,n,d_{\max}),$$

where

(4) $$\begin{array}{c} \begin{array}{c} \left|\Sigma {|}^{k}\gg (l-k+1\right) \end{array}\\ \begin{array}{c} (\left\lfloor l/n\right\rfloor -k+1)\cdot n>d_{\max}\cdot k\\ 1\leq n\leq d_{\max} \end{array} \end{array}.$$

The probability is derived under the assumption that these $d_{\max}$ bases are evenly distributed in two or more similar sequences. In real NGS data, these shifting bases are not evenly distributed because they are errors or mutations originating from the same or similar molecular generated during the PCR and sequencing processes. Additionally, when the threshold $d_{\max}$ is set as a bigger number (e.g. 5), k will become quite small which conflicts with the conditions above and may lead to quite large-sized bins after bucketing. To obtain a reasonable bucket size and number k and an optimal balance between w and k, we only consider at most three differential bases within a segment which can be given by

(5) $$\frac{3k}{(\left\lfloor l/n\right\rfloor -k+1)\cdot n}<p_{t},$$

where $p_{t}$ is a predetermined threshold probability. By predefining a probability $p_{t}$ and determining a segmentation number n based on the read length l, we can estimate the k-mer length k and set the window size as w=⌊α*⌊l/n⌋⌋, where 0<α≤1.

#### 2.3.2 Bucketing via one-minimizer-one-read with random minimizer

We apply the estimated value of k from the multi-minimizer-one-read with random minimizer approach to the one-minimizer-one-read with random minimizer bucketing mode. The rationale is that, in the multi-minimizer-one-read approach, reads are divided into equal-sized segments, resulting in a consistent k value across segments. Since k is valid within each segment, it remains applicable for the entire read. One key difference is that in the segmented approach, the last k-mer of each segment (except for the final segment) is considered only once during minimizer sampling, whereas without segmentation, these substrings are taken into account multiple times as the sliding window moves across the read. Once k is determined, the value of w can be calculated based on the segment size, noting that the number of selected k-mers increases as the window size decreases.

#### 2.3.3 Bucketing via one-minimizer-one-read with Miniception

In the original Miniception study (Zheng *et al.* 2020), the specific density of a minimizer on a sequence S is defined as the expected number of selected k-mer divided by the total number of k-mers in the sequence S, and Miniception guarantees a density of <2/(w+1). We apply this expected density and its upper bound to estimate the k-mer length for bucketing short reads when using Miniception. Although this bound was originally derived for long sequences, our empirical tests on short reads suggest that the minimizer density remains effective. This observation allows us to approximate the minimizer density as

(6) $$\begin{array}{c} \frac{m}{l-k+1}\approx \frac{2}{w+1}\\ w=\left\lceil \beta \cdot k\right\rceil \end{array},$$

where m is the number of expected k-mers selected from a read of length l, β is factor used to determine the window size based on the k-mer length.

To test the bucketing performance of multi-minimizer-one-read approach with Miniception in our heuristic pipeline, we estimate k using the formula (6), substituting the segment size ⌊l/n⌋ (after segmentation) for the read length l and the expected number of k-mers as (⌈m/n⌉) for each segment.

After generating minimizers for all the reads in R, we assign every read to a unique bin based on their k-mers. Then a pairwise edit distance calculation is performed for the reads within normal-sized buckets in parallel. Read pairs whose edit distance falls in the predetermined interval $\left[d_{\min},d_{\max}\right]$ are inserted to the graph as edges, and the edit distance is attributed as the edge weight. For those large bins, OMH with gapped k-mers is used to bucket them again.

### 2.4 Re-bucketing by an order min hash with gapped k-mers

We proposed an Order Min Hash with gapped k-mers (gOMH) to partition these large-sized buckets. Our idea gOMH is based on the original OMH (Marçais *et al.* 2019) that is explicitly designed for edit distance calculation and is sensitive to the relative order of the k-mers in the reads. The original OMH sketch involves counting the occurrence of each k-mer in a sequence and then using both the k-mer and its occurrence for hashing. This operation requires looping twice over the k-mer set of each read, and the occurrences obtained for the same k-mer are identical. In our approach, we loop over the set of k-mers only once and utilize the current k-mer frequency as additional information for hashing. This approach reduces time complexity and increases the diversity of hash values. Notably, our method produces unique hashes even for repeated k-mers, eliminating the need for partial sorting of hashes and position sorting as required in the original OMH. Additionally, gOMH is designed and applied for specific scenarios in this study, and the increased hash value diversity may impact the collision probability, potentially limiting its applicability to other contexts.

As these reads grouped in the large-sized buckets are generated after minimizer-bucketing, they have at least one same substring in common. Therefore, we random select a gapped k-mer with the minimum hash value from a read to represent this read. The gapped k-mer here defines a substring that constitutes with bases orderly selected from a read by skipping one base every two positions. For example, ‘ACA’ (‘A-C-A’, ‘-’ refers to a gap) and ‘GTG’ (‘G-T-G’) are two gapped 3-mers of the sequence ‘AGCTAGT’. For a read r with length l and k-mer size $k^{\prime }$, there are $l-2k^{\prime }+2$ gapped k-mers in r. Therefore, the probability of selecting an unmutated gapped k-mer from one read to represent this read is

(7) $$p_{2}\geq \frac{l-2k^{\prime }+2-d_{t}\cdot k^{\prime }}{l-2k^{\prime }+2},$$

where $d_{t}\in [d_{\min},d_{\max}]$. Therefore, given l and $d_{t}$ with a predefined probability $p_{2}$, the k-mer size is given by

(8) $$\begin{array}{c} k^{\prime }=\frac{\left(l+2)\cdot (1-p_{2}\right)}{2+d_{t}-2p_{2}} \end{array}.$$

For each $d_{t}$ in $\left[d_{\min},d_{\max}\right]$, we determine a k-mer size and generate a random seed as well. If $d_{\max}$ is less than a threshold (e.g. 3), we repeat the permutations with the threshold times to ensure sufficient permutations for bucketing. The pseudocode of gOMH bucketing is presented in Supplementary Algorithm S1, while our gOMH calculation for a read is outlined in Supplementary Algorithm S2. The complete process of gOMH bucketing for a set of reads is described below.

- Calculating the k-mer size $k^{\prime }$ using Equation (8) for different $d_{t}$ and generating a random seed each time as well.
- Iteratively calculating the gOMH value with a pair of *seed* and $k^{\prime }$ for all the reads in R. For each read r, we
  - looping over all the gapped k-mers and storing the hash value *val* by combined hashing the current seed, the gapped k-mer and its current occurrence number.
  - finding the minimal hashing value *val* as gOMH value.
- Using each gOMH value to represent a read once. Similar reads with the same gOMH value will be grouped in one bucket.

After assigning the reads in the large-sized buckets binned by minimizer-bucketing using our gOMH bucketing, the edit-distance graph can be once again updated with a process similar to the one after minimizer-bucketing.

### 2.5 New edges detected by neighbourhood traversal

After the above two-layer bucketing and edge detection within each bucket, a majority of the required edges can be found. However, there may still exist large-sized bins that cannot be divided into smaller subgroups using minimizers or the gOMH-bucketing. We use a neighbourhood traversal process for these large-sized bins.

Specifically, for each remaining read, we use a recursive process to visit its neighbours and neighbours’ neighbours, selecting nodes that may have a small edit distance with the target node. We then calculate the actual edit distance between these candidate node pairs. The rationale behind this approach is rooted in our observation that in a complete edit-distance graph (i.e. a read graph with all the edit-distance edges falling in an interval connected), certain relationships adhere to the triangle inequality principle:

(9) $$\begin{array}{c} \begin{array}{c} e(x,y)+e(y,z)\geq e(x,z) \end{array}\\ \begin{array}{c} e(x,y)+e(x,z)\geq e(y,z)\\ e(y,z)+e(x,z)\geq e(x,y) \end{array} \end{array},$$

where e(x,y), e(x,z) and e(y,z) represent the weight between the nodes x and y, x and z , and y and z, respectively. Note that while we do not use the triangle inequality principle to determine edge weights, it provides the rationale and motivation for using the traversal process.

**Figure 2.**
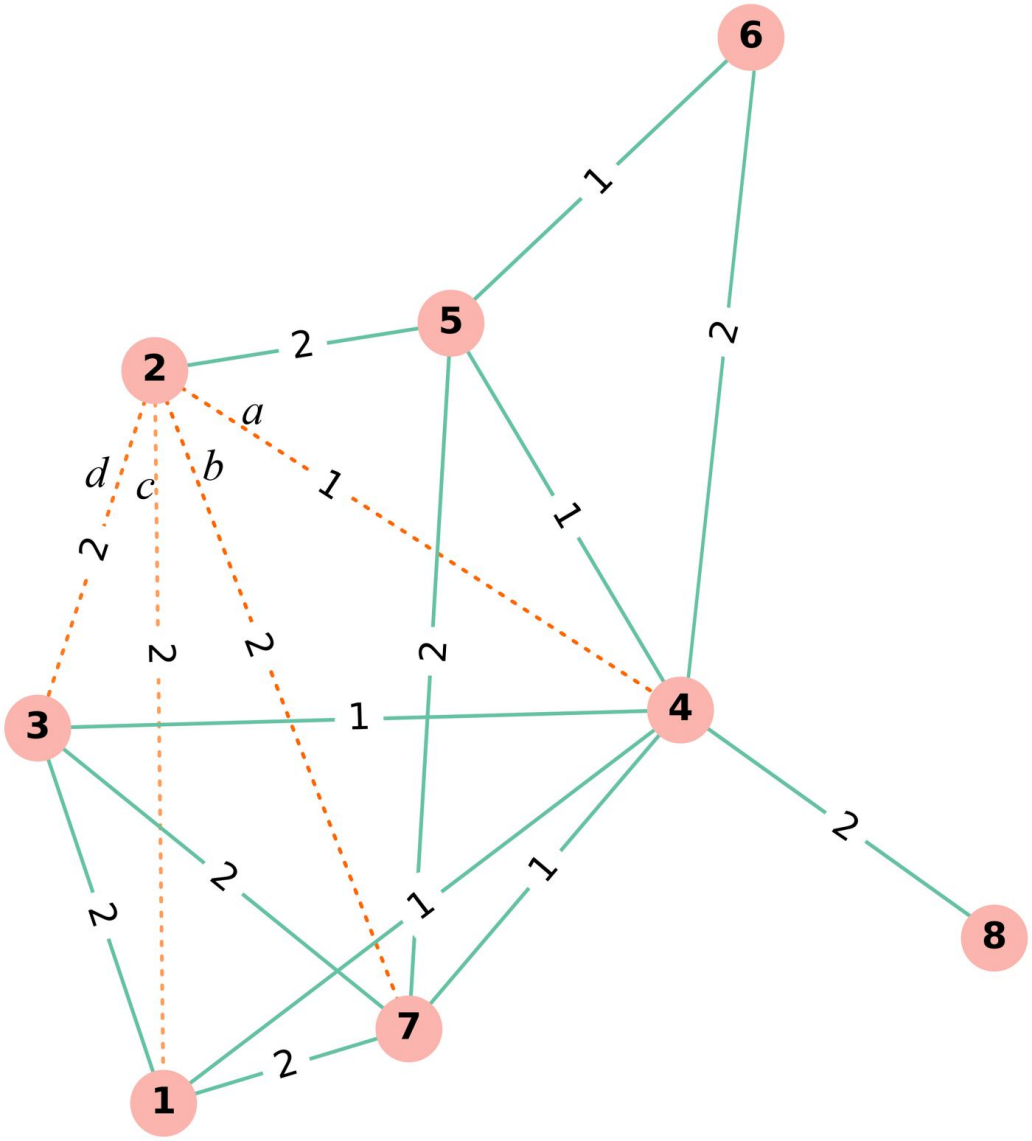
An example of subgraph illustrating how neighbourhood traversal recovers edges. The dashed edges of *a*, *b*, *c*, and *d* can be discovered by visiting node 2’s neighbourhood within 2–4 hops.

As the currently constructed graph may not always be a complete edit-distance graph, we visit those nodes that are multiple times the maximum edit distance (e.g. 3 * $d_{\max}$) away from a given node to uncover potential edges. The pseudocode for updating the graph using this traversal approach is provided in Supplementary Algorithm S3. As an illustrative example, node pairs connecting the edges *a*, *b*, *c*, *d* can be discovered via a graph traversal by visiting two to four neighbouring nodes of node 2 (Fig. 2), and their real edit distance can be further determined.

Furthermore, to construct a graph with edge weights falling within the range of $\left[d_{\min},d_{\max}\right]$ where $d_{\min}>1$, our method involves identifying all edges within the weight range of $\left[1,d_{\max}\right]$. This facilitates the discovery of additional edges with larger edit distances through those with smaller edit distances. Once the graph construction is completed, edges within the weight range of $\left[1,d_{\min}\right)$ will be removed.

### 2.6 Special considerations in the construction of UMI graphs of edit distances

The length of UMIs typically ranges from 8 to 12 bases. Using two windows for bucketing UMIs yields only 1 to 3 k-mer candidates per window when k=4. The multi-minimizer-one-window bucketing method is ineffective for a large number of unique UMIs (e.g. >1 million). Therefore, we opt for one-minimizer-one-read bucketing with random minimizer and Miniception for UMIs. Since a single round may miss many potential edges, reads2graph incorporates an additional round of bucketing using the gOMH approach and graph traversal for unique sequences in normal bins generated by minimizers. Moreover, if the number of gOMH permutations is not less than the number of k-mer candidates, random selection will utilize the same k-mer as the minimum hashing value for bucketing. In such cases, to reduce unnecessary computations, reads2graph uses all k-mer candidates for bucketing instead of resorting to random selection and hashing value calculation.

## 3 Results

The performance of reads2graph was evaluated on four single-end sequencing datasets (ERR187525, ERR187844, SRR1543964, and SRR1543965), two paired-end sequencing datasets (SRR9077111 and SRR22085311) and four sets of UMI sequences extracted from SRR1543964, SRR1543965, SRR28313990, and SRR28314008. The length of the short reads ranges from 18 to 238 in these datasets; and the length of the UMIs is 11 or 12.

### 3.1 Performance for building edit-distance graphs from short reads

To assess the performance of reads2graph, we evaluate the completeness of its identified edges by comparing the number of edges in the edit-distance graphs generated by reads2graph to those in the ground truth graphs. To our knowledge, reads2graph is the first software designed to construct an edit-distance graph from a short-read set. Therefore, to provide a comprehensive evaluation of its efficiency, we also implemented two additional baseline methods for bucketing to construct the edit-distance read graph: one solely using Miniception (Zheng *et al.* 2020), and the other solely using the original OMH (Marçais *et al.* 2019) for bucketing. In the experimental settings, various edge weight intervals *[1, j]*, 1≤j≤5, are used, where every interval defines an edit-distance threshold j for the edges of the graphs.

The average completeness for different edit-distance intervals, comparing reads2graph in four different modes with baseline methods, is presented in Table 1. The corresponding average running time and memory usage are shown in Table 2. Supplementary Tables S1–S3 present the detailed performance of reads2graph in terms of completeness, runtime, and memory usage across various edit-distance intervals, respectively. These results, obtained using different minimizer bucketing modes, are compared with baseline methods including Miniception-only, OMH-only bucketing, and the brute force approach.

**Table 1.** The average completeness for different edge weight intervals in constructing an edit-distance graph using reads2graph across four bucketing modes: random minimizer with or without read segmentation, and Miniception with or without segmentation.^a^

Datasets	Read length	No. of total reads	No. of unique reads	Methods					
				Miniception-only	OMH-only	reads2graph^b^	reads2graph^c^	reads2graph^d^	reads2graph^e^
ERR187525	18–36	7 300 933	746 407	95.94%	44.60%	95.10%	95.48%	94.63%	95.08%
ERR187844		5 885 262	602 347	97.94%	44.39%	95.11%	95.39%	95.09%	95.00%
SRR9077111_1	101	4 271 222	742 130	98.96%	46.36%	100.00%	99.89%	99.96%	99.05%
SRR9077111_2		4 271 222	739 903	98.07%	52.19%	99.57%	98.39%	96.71%	85.95%
SRR22085311_1	151	3 599 812	2 131 269	99.92%	84.25%	97.69%	99.06%	97.45%	93.90%
SRR22085311_2		3 599 812	2 042 003	99.92%	81.42%	99.77%	99.72%	99.97%	99.90%
SRR1543964	238	1 100 818	312 070	100.00%	88.03%	99.88%	99.98%	99.94%	99.91%
SRR1543965		952 554	273 707	100.00%	87.61%	99.93%	99.99%	99.95%	99.93%
Average				98.84%	66.11%	98.38%	98.49%	97.96%	96.09%

a The results are compared to those obtained using solely Miniception and OMH bucketing methods.

b Using random minimizer with read segmentation as minimizer bucketing.

c Using random minimizer without read segmentation as minimizer bucketing.

d Using Miniception without read segmentation as minimizer bucketing.

e Using Miniception with read segmentation as minimizer bucketing.

**Table 2.** The average running time (*T*) in minutes and memory (*M*) usage in megabytes for different edge weight intervals in constructing an edit-distance graph using reads2graph across four bucketing modes: random minimizer with or without read segmentation, and Miniception with or without segmentation.^a^

Datasets	Read length	No. of total reads	No. of unique reads	Methods													
				Brute force		Miniception-only		OMH-only		reads2graph^b^		reads2graph^c^		reads2graph^d^		reads2graph^e^	
				*T*	*M*	*T*	*M*	*T*	*M*	*T*	*M*	*T*	*M*	*T*	*M*	*T*	*M*
ERR187525	18–36	7 300 933	746 407	996.7	2254	20.0	1752	4.4	951	133.8	5270	75.6	4062	45.3	3126	140.7	3890
ERR187844		5 885 262	602 347	645.0	4746	21.1	2566	4.6	1084	157.3	5519	85.2	4434	46.1	3614	128.7	4190
SRR9077111_1	101	4 271 222	742 130	1629.3	3049	308.6	2854	812.1	1574	227.3	34 826	59.8	11 712	53.3	7211	43.2	5133
SRR9077111_2		4 271 222	739 903	2040.5	3142	1140.0	2785	1184.0	1648	199.9	31 858	66.4	8239	65.6	8136	47.8	7433
SRR22085311_1	151	3 599 812	2 131 269	7804.4	8894	2571.3	8893	351.4	6104	164.2	18 809	208.4	20 129	198.7	23 851	79.7	15 347
SRR22085311_2		3 599 812	2 042 003	7110.1	3961	2964.9	7114	134.4	4870	150.0	15 718	58.2	14 523	120.4	24 152	124.3	13 543
SRR1543964	238	1 100 818	312 070	317.7	1555	278.9	2192	844.8	1324	91.5	7021	21.9	3234	19.5	3544	20.7	3195
SRR1543965		952 554	273 707	240.8	1302	190.9	1889	629.2	1134	70.5	5645	17.0	2940	15.3	3175	16.8	2945
Average				2598.0	3613	937.0	3756	495.6	2336	149.3	15 583	74.1	8659	70.5	9601	75.2	6960

a These results are compared to the baseline methods: Miniception-only bucketing, OMH-only bucketing and brute force. Experiments used 56 CPU cores for ERR187525 and ERR187844, and 64 for other datasets, with all methods using consistent cores per dataset.

b Using random minimizer with read segmentation as minimizer bucketing.

c Using random minimizer without read segmentation as minimizer bucketing.

d Using Miniception without read segmentation as minimizer bucketing.

e Using Miniception with read segmentation as minimizer bucketing.

Overall, reads2graph in different minimizer bucketing modes achieves high average completeness, ranging from 94.63% to 100%, except for the Miniception with read segmentation mode on datasets SRR9077111_2 and SRR22085311_1 (see Table 1). Specifically, for datasets with equal-length sequences, our method—excluding the Miniception with read segmentation mode—achieves completeness between 99% and 100% in most cases, with only four exceptions where completeness ranges from 96.71% to 98.39% (see Table 1). These results demonstrate that our edit-distance graph construction method misses only a minimal number of ground-truth edges and, in some cases, captures all of them. Among the different modes, reads2graph using random minimizer or Miniception without read segmentation achieves the best balance between completeness and running time, followed by the mode with random minimizer based on read segmentation.

The baseline method using Miniception-only bucketing achieves high completeness across all datasets, while OMH produces the worst results among all methods. Notably, for datasets with read lengths ranging from 18 to 36, Miniception-only bucketing outperforms reads2graph while requiring less running time. However, the Miniception-only method incurs significantly longer runtimes, consuming 33% (2571.3 min), 42% (2964.9 min), and 56% (1140 min) of the brute-force method’s runtime on the large datasets SRR22085311_1, SRR22085311_2, and SRR9077111_2, respectively, using 64 CPU cores in each case. In contrast, three modes of reads2graph achieve comparable completeness to Miniception-only while requiring significantly less running time. For instance, reads2graph with random minimizer bucketing, without read segmentation, requires only 5.8% (66.4 min) of the runtime of the Miniception-only method (1140 min) and 3.3% of the runtime of the brute-force approach (2040.5 min) on the SRR9077111_2 dataset. Despite its efficiency, reads2graph achieves a completeness of 98.39%, slightly higher than Miniception’s 98.07%. Regarding memory usage, reads2graph consumes more memory than baseline methods; however, the required memory is typically manageable on modern high-performance computing clusters.

**Figure 3.**
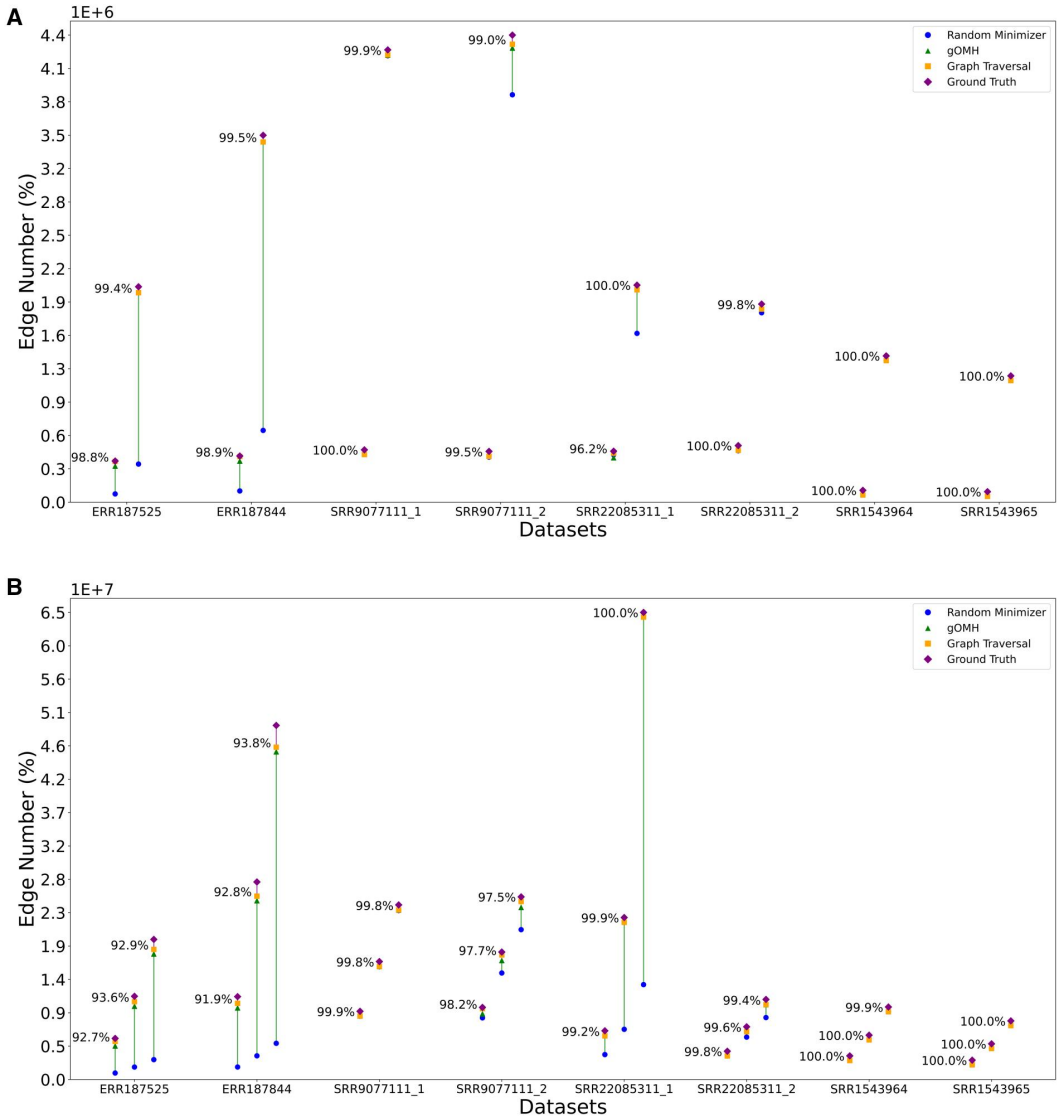
Lollipop plot depicting the progression of performance for reads2graph across multiple datasets and intervals, with each marker representing a different stage: Random minimizer bucketing without read segmentation, gOMH bucketing, and Graph Traversal. The *y*-axis shows the number of edges constructed by reads2graph, with the percentage relative to the ground truth labelled next to the final-stage marker. In subplot (A), intervals [1,1] (left) and [1,2] (right) are used for each dataset, while subplot (B) features intervals [1, 3], [1, 4] and [1, 5] from left to right for each dataset. gOMH: gapped *k*-mer based Order Min Hash. In the experimental settings of reads2graph, a bucket is classified as a large bucket if it contains 10 000 or more reads.

To investigate the effect of each stage of reads2graph contributed the final completeness, we draw lollipop plots to show the progressed completeness. Two lollipop plots in Fig. 3 illustrate the completeness performance of reads2graph in detecting edges using random minimizer bucketing without read segmentation, with ablation results summarizing the graph construction status across the three stages. Supplementary Figs S1–S3 present the progressed completeness achieved using the random minimizer with read segmentation, as well as Miniception with and without read segmentation. These results indicate that edge completeness is progressively attained through different stages. This trend is particularly evident in certain datasets, such as ERR187525, ERR187844, and SRR22085311. In contrast, reads2graph achieves high completeness immediately after the graph is updated through the minimizer bucketing process, and the completeness is further improved after updating graph with gOMH bucketing. In rare instances, the constructed graph undergoes an additional update through graph traversal (see Supplementary Tables S1 and S2). Nonetheless, graph traversal remains essential, as it ensures the thorough processing of all buckets generated by minimizer and gOMH.

The multi-minimizer-one-read strategy (i.e. segmenting each read into independent parts) with random minimizer bucketing achieves high average completeness (see Table 1), demonstrating the effectiveness of the read segmentation approach when combined with random minimizer bucketing. However, it is not the most efficient in terms of balancing speed and completeness, as its running time is approximately double that of random minimizer bucketing without segmentation (see Table 2). One possible reason is that the window size in the multi-minimizer-one-read strategy is smaller than the window size used without read segmentation in the experiments conducted in this study. The multi-minimizer-one-read strategy on Miniception shows limited effectiveness, working only on certain datasets. For example, the Miniception bucketing with read segmentation mode achieves high completeness (99%) with the least running time among all methods.

### 3.2 Edit-distance graphs constructed from UMI sequences

The number of edges and our edge detection completeness in the construction of the edit-distance graph from the UMI sequences are presented in Table 3 in comparison with the ground truth.

**Table 3.** The performance in terms of completeness, running time (min) and memory usage (megabytes) in the construction of edit-distance graphs by reads2graph with random minimizer and Miniception bucketing without read segmentation from the UMI sequences under the edge weight intervals [1, 1] and [1, 2].^a^

Datasets		SRR1543964		SRR1543965		SRR28314008		SRR28313990	
UMI length		12				11			
No. of total UMIs		1 119 301		986 833		7 563 556		5 943 780	
No. of unique UMIs		99 727		101 557		853 590		1 356 538	
Edge weight intervals		[1, 1]	[1, 2]	[1, 1]	[1, 2]	[1, 1]	[1, 2]	[1, 1]	[1, 2]
Brute force	No. of edges	196 559	2 286 391	95 262	803 724	3 707 160	76 027 038	8 007 796	168 020 246
	Time	11.0	11.7	11.1	11.6	1091.1	1157.8	2353.9	2836.0
	Memory	85	268	74	141	867	7403	1553	16 067
reads2graph^b^	No. of edges	196 559	2 273 764	95 262	797 329	3 531 448	74 004 379	7 627 205	162 800 754
	Completeness	100.00%	99.45%	100.00%	99.20%	95.26%	97.34%	95.25%	96.89%
	Time	2.3	3.4	2.1	3.1	107.7	346.9	237.9	737.8
	Memory	224	428	214	300	2797	9048	4440	18 503
reads2graph^c^	No. of edges	194 641	2 222 963	94 364	778 839	3 531 510	73 471 898	7 627 261	162 361 154
	Completeness	99.02%	97.23%	99.06%	96.90%	95.26%	96.64%	95.25%	96.63%
	Time	2.5	2.7	1.4	2.4	85.1	271.4	218.1	641.5
	Memory	200	403	192	277	2606	8842	4187	18 225

a Experiments used 56 CPU cores for SRR1543964 and SRR1543965, and 64 for SRR28314008 and SRR28313990, with all methods using consistent cores per dataset.

b Using random minimizer without read segmentation as minimizer bucketing.

c Using Miniception without read segmentation as minimizer bucketing.

Our method reads2graph, in both Miniception and random minimizer bucketing modes, detected a very high percentage of ground truth edges in less than half the running time of the brute-force method on SRR1543964 and SRR1543965, each containing approximately 100 000 unique UMI sequences. As the number of unique UMIs increased to around one million, reads2graph still achieved a high detection rate (95.25%–97.34%) while being 4 to 13 times faster than brute-force solutions.

### 3.3 Slight performance varies when the large-bucket size-threshold changes

To investigate the impact of the large-bucket size-threshold on the algorithm performance, we set different large-bucket size-thresholds for building edit-distance graphs from SRR9077111_1 and SRR9077111_2, SRR1543964, and SRR1543965. We define the change in running time and memory as the difference between their respective results under different thresholds and those under the threshold 10 000. Additionally, we define the change of completeness in percentage ($pct_{c}$) as the ratio of the change in the number of edges to the ground truth value, calculated by

(10) $$pct_{c}=\frac{E_{x}-E_{10 000}}{E_{\mathrm{truth}}}$$

where $E_{x}$ represents the total number of edges when the threshold is x and $E_{\mathrm{truth}}$ is the number of total ground truth edges.

**Figure 4.**
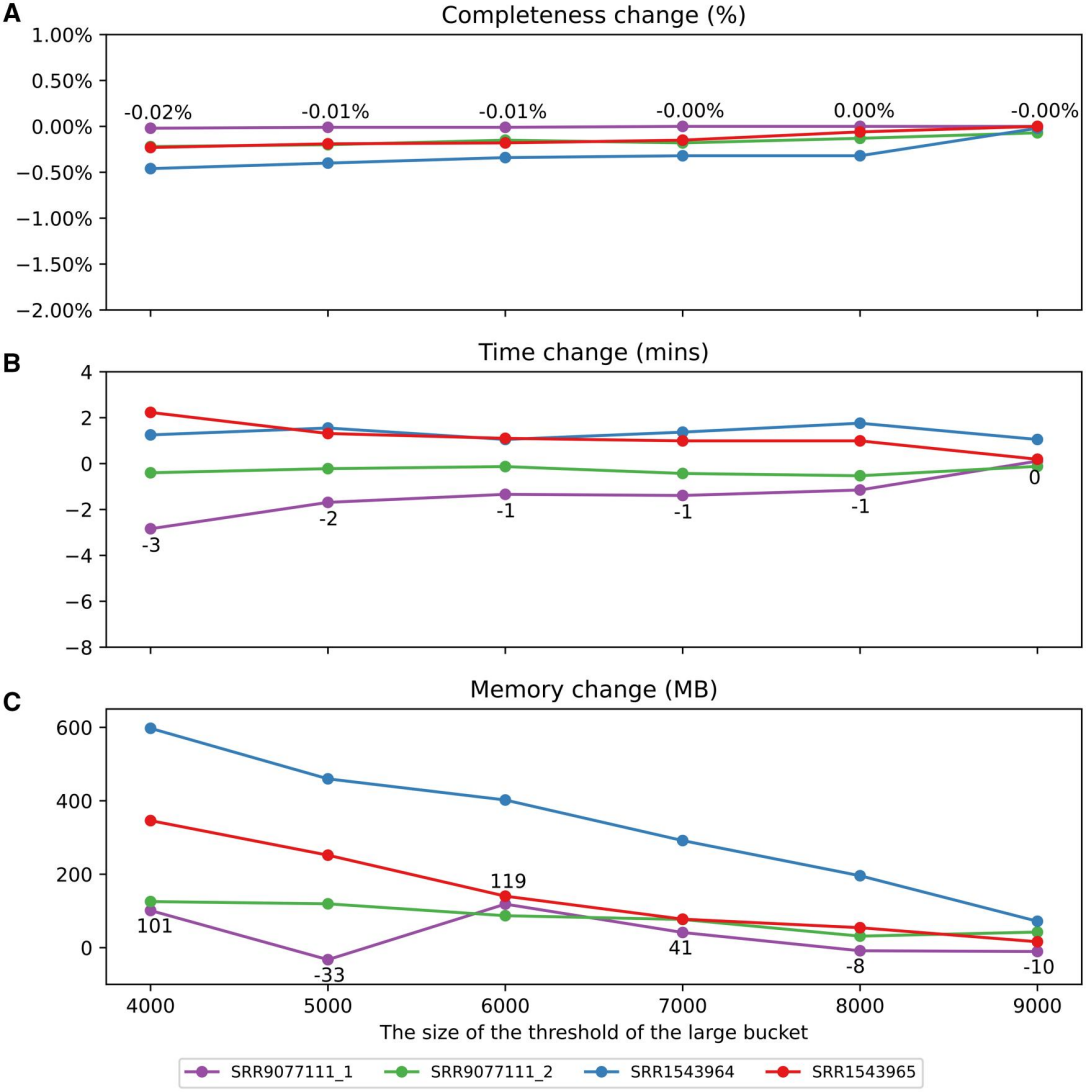
Performance of reads2graph using random minimizer bucketing without read segmentation at varying large bucket thresholds. Metrics include changes in completeness (A), runtime (B), and memory usage (C) when constructing read graph with the edit distance interval set to [1, 3].

Reads2graph can capture nearly all edges with edit distances within the range [1, 3] when the threshold size of the large bucket is set at 10 000, as demonstrated in Fig. 3. We calculated the changes in completeness percentage, runtime, and memory consumption for the edit distance interval [1, 3] using different size-thresholds of the large buckets. And the results for various minimizer bucketing modes—random minimizer without read segmentation, random minimizer with read segmentation and Miniception bucketing without read segmentation—are presented in Fig. 4, Supplementary Figs S4 and S5, respectively.

Across the results for three modes on the four datasets, the completeness percentage of edges shows slight fluctuations, particularly in the two modes using random minimizer bucketing (see Fig. 4 and Supplementary Fig. S4). The largest decrease, 2.16%, occurs at a threshold of 4000 on the SRR1543964 dataset for the Miniception bucketing mode without segmentation (Supplementary Fig. S5). However, its completeness remains high, ranging from 95.7% to 96.6%. As the threshold increases, performance changes across all datasets become increasingly negligible.

Regarding runtime and memory consumption, reads2graph completes the task with minimal variation across different thresholds, except for runtime when using Miniception without read segmentation on the SRR9077111_2 dataset. However, even in this case, the longest runtime is 81.9 min at a threshold of 10 000 (See Supplementary Table S2). Overall, the impact of the large bucket size threshold on algorithm performance is negligible. These results suggest that reads2graph performs well with the default large bucket size threshold.

## 4 Discussion

Bucketing reads via minimizer and gOMH in parallel without detecting precise edit-distance-based read relationships is quick but may result in information (edge) loss and redundant usage of read pairs across different bins. In contrast to the steps of bucketing reads, constructing a complete edit-distance read graph is more time-consuming due to the following reasons. Inserting edges is not thread-safe and must be executed sequentially, even if other processes are available. As the right boundary of the edit distance interval increases, the number of edges and the time required for insertion grow substantially. Additionally, some edit distance calculations are redundantly performed in different buckets for the same sequence pairs. To avoid repeated calculations, existence checks must precede edit distance calculations. However, these checking steps cannot be parallelized as well, and their time complexity is similar to that of inserting edges.

One time-consuming aspect of constructing the edit-distance graph is identifying edges within large buckets. In this study, our primary goal with the bucketing strategy is to maximize completeness by grouping as many similar reads as possible into the same bucket; therefore, we did not investigate minimizing false negatives (i.e. read pairs with large edit distances that are hashed to the same bucket). This approach may result in some dissimilar reads being placed in the same bucket, particularly when constructing small edit-distance read graphs from real sequencing datasets. In future work, we will explore strategies to maximize true positives while minimizing false negatives in bucketing to further enhance the performance of reads2graph.

The time complexity of the graph traversal is determined by three factors: the number of unique reads ($\left|R_{sub\_x}\right|$) from the large-size buckets, the maximum edit distance (or traversal depth) ($d_{\max}$), and the maximum node degree (δ). As a result, the time complexity of the traversal is expressed as $O(|R_{sub\_x}|\cdot \delta^{d_{\max}})$. In cases involving short reads (typically less than 300 bp) where the maximum edit distance $d_{\max}$ is small, the actual node degree (δ) is limited by both the small edit-distance threshold and biological constraints. Under these conditions, the graph traversal’s time complexity is primarily influenced by the number of unique reads in the large-sized buckets.

Finding the optimal parameters to efficiently identify all small edit-distance-based edges from a set of short reads is challenging, as it involves balancing completeness and efficiency. This study offers heuristic methods to determine optimal parameters to achieve a high completeness of edit-distance edge detection for large-scale datasets of short reads while maintaining time and memory efficiency. The proposed method can be applied to various bioinformatics applications, including data compression, error correction, deduplication and theoretical analysis of novel minimizer and LSH schemes. In future work, we will apply reads2graph to explore its benefits in these downstream or relevant analyses.

## 5 Conclusion

This study introduced an efficient heuristic method (reads2graph) for constructing edit-distance graphs from large-scale short-read sequencing datasets. The method has three sides of novelties or contributions. First, we investigate whether the completeness of edge detection can be improved using a multi-minimizer-one-read bucketing strategy with both random minimizer and Miniception. By optimizing the k-mer size and window size, our method achieves high completeness of edge detection while maintaining computational efficiency. Second, we propose the gOMH bucketing method with optimized parameters to better partition large buckets, allowing more edges to be detected. Third, we introduce a graph traversal step under the triangle inequality principle to identify additional edges within large, previously unbucketed subgroups of reads. Extensive experimental results demonstrate that reads2graph achieves near-complete edge detection, approaching 100% in most cases, while significantly outperforming brute-force edge detection methods in speed. Moreover, compared with solely using the bucketing method of Miniception or Order-Min-Hash, reads2graph achieves an excellent balance in completeness and efficiency.

## Supplementary Material

vbaf081_Supplementary_Data

## Data Availability

The sequencing data used in this study were downloaded from SRA under accessions SRR1543964, SRR1543965, SRR9077111, SRR22085311, SRR28313990, and SRR28314008, and from ENA under accessions ERR187525 and ERR187844.
