# Supplementary material for: Construction of edit-distance graphs for large sets of short reads through minimizer-bucketing
Source: Bioinform Adv. 2025 Apr 10;5(1):vbaf081. doi: 10.1093/bioadv/vbaf081 (PMC12040381; doi:10.1093/bioadv/vbaf081)
Supplement: vbaf081_Supplementary_Data [file vbaf081_supplementary_data.zip › SupplementaryInformation.pdf]

Supplementary Information
for
Construction of edit-distance graphs for large sets of short reads through minimizer-bucketing

April 4, 2025

Supplementary Algorithm 1 Bucketing reads with gOMH

function GOMH2READ(Vector R, Int l, Int T, Int d_{max}, Float p_2)
    seeds_k ← empty vector; $d_t \leftarrow d_{max}$
    $t \leftarrow (T$ if $d_{max} \leq 2$ else $1)$
    for cur_d ← d_t **downto** 1 **do**
        for $j \leftarrow 0$ **to** t **do**
            seed ← random number within desired range
            $k' \leftarrow \lceil \frac{(1-p_2)\times(2+l)}{d_t+2-2*p_2} \rceil$; if $k' < 4$, then $k' \leftarrow 4$; else if $k' > 27$, then $k' \leftarrow 27$.
            pair ← $(seed, k')$
            seeds_k.push_back(pair)
        end for
    end for
    for r in R **do** ▷ Parallelize this loop with OpenMP
        for $[seed, k']$ in seeds_k **do**
            $v \leftarrow gOMH(r, k', seed)$
            $gomh2reads[v] \leftarrow r$
        end for
    end for
    return $gomh2reads$
end function

Supplementary Algorithm 2 gOMH calculation for a read

```
function gOMH(Read r, Int k', Int seed)
    if |r| < 2 * k' − 1 then return
    end if
    cur_seed ← seed
    for i ← 0 to |r| − 2 * k' + 1 do
        kmer ←''; j ← i; count ← 0
        while j < |r| − 2 * k' + 2 ∧ count < k' do
            kmer ← kmer + r[j]
            count ← count + 1
            j ← j + 2
        end while
        occ ← occurrence[kmer] + +
        boost :: hash_combine(cur_seed, kmer)
        boost :: hash_combine(cur_seed, occurrence[kmer])
        hashes.emplace_back(cur_seed)
        cur_seed ← seed
    end for
    return std :: min_element(hashes)
end function
```

Supplementary Algorithm 3 Update graph with graph traversal

function UPDATEGRAPHWITHTRAVERSAL(Graph G, Vector S_r, Int T_{dep}, Int d_{max})
    $P_v \leftarrow \emptyset$ ▷ Store vertex pairs
    for all $r \in S_r$ **do** ▷ Parallelize this loop with OpenMP
        $v_r \leftarrow$ read2vertex(r) ▷ Map read to vertex
        $N_{v_r} \leftarrow$ VisitNeighborsWithThreshold(G, v_r, T_{dep})
        for all $v_i \in N_{v_r}$ **do**
            $P_v \leftarrow P_v \cup (v_r, v_i)$
        end for
    end for
    for all $(v_j, v_k) \in P_v$ **do** ▷ Parallelize this loop with OpenMP
        $r_j \leftarrow$ vertex2read(v_j)
        $r_k \leftarrow$ vertex2read(v_k)
        $e_d \leftarrow$ edit_distance(r_j, r_k)
        if $1 \leq e_d \leq d_{max}$ **then**
            $G.E \leftarrow G.E \cup (r_j, r_k, e_d)$ ▷ Add edge (r_j, r_k) with e_d to graph G
        end if
    end for
end function

function VISITNEIGHBORSWITHTHRESHOLD(Graph G, Vertex v_r, Int T_{dep})
    $N_{v_r} \leftarrow \emptyset$ ▷ Stores neighbors within threshold T_{dep}
    $flag[v] \leftarrow \text{false}, v \in V$ ▷ Mark all nodes unvisited
    Visit(G, v_r, T_{dep}, 0, N_{v_r}, $flag$)
    return N_{v_r}
end function

function VISIT(Graph G, Vertex v_r, Int T_{dep}, Int C_{dep}, Vector N_{v_r}, Vector $flag$)
    $flag[v_r] \leftarrow true$ ▷ Mark current node as visited
    for all $v_k \in$ adjacent_vertices(v_r, G) **do**
        if not $flag[v_k]$ and $C_{dep} + 1 \leq T_{dep}$ **then**
            $N_{v_r} \leftarrow N_{v_r} \cup v_k$ ▷ Add neighbor to the list
            Visit(G, v_k, T_{dep}, $C_{dep} + 1$, N_{v_r}, $flag$) ▷ Recursive visit
        end if
    end for
end function

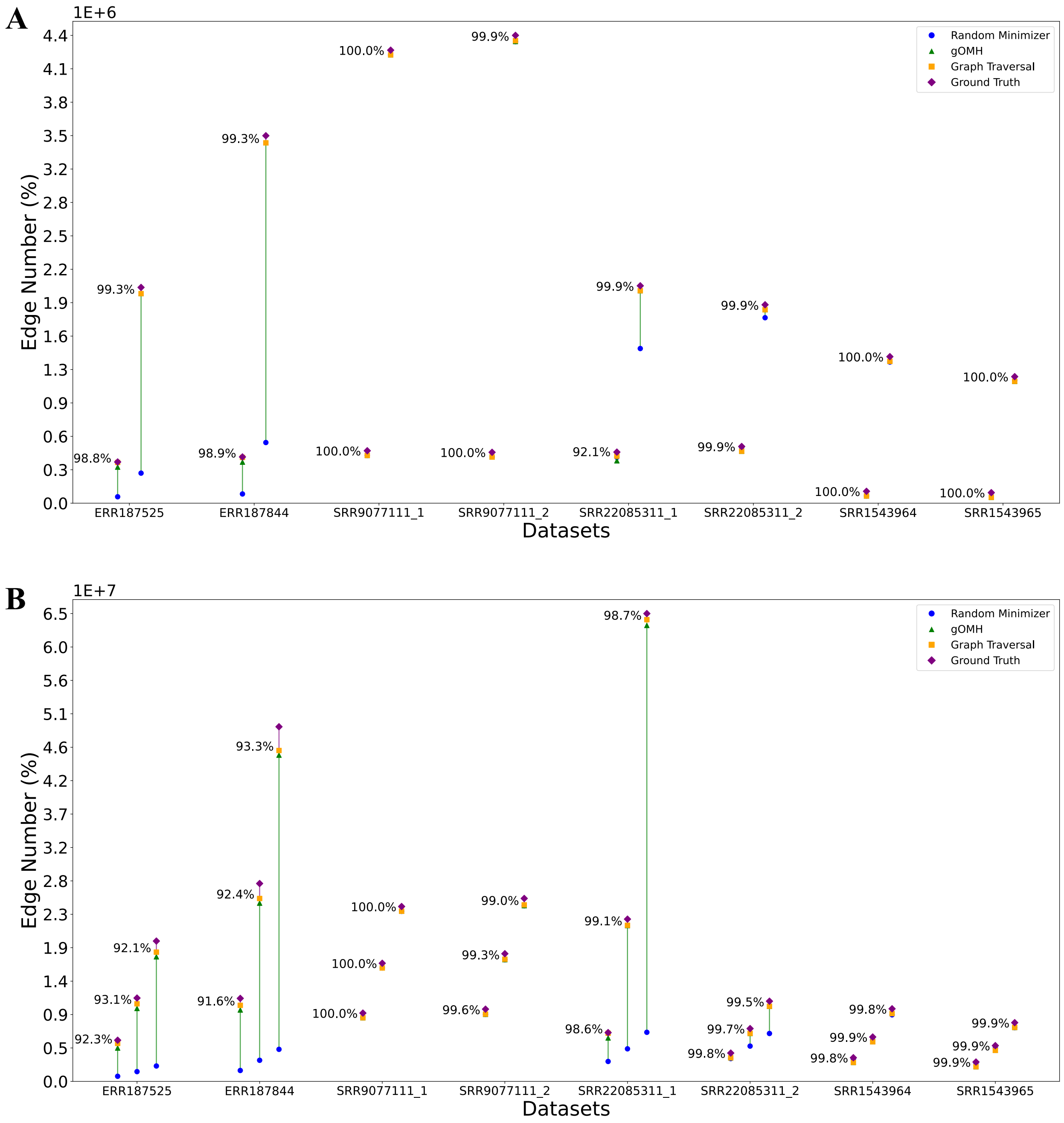

Supplementary Figure 1: Lollipop plot depicting the progression of performance for reads2graph across multiple datasets and intervals, with each marker representing a different stage: Random minimizer (blue) bucketing with read segmentation, gOMH (green) bucketing, and Graph Traversal (orange). The y-axis shows the number of edges constructed by reads2graph, with the percentage relative to the ground truth labeled next to the final-stage marker. In subplot (**A**), intervals [1,1] (left) and [1,2] (right) are used for each dataset, while subplot (**B**) features intervals [1, 3], [1, 4] and [1, 5] from left to right for each dataset. gOMH: gapped k-mer based Order Min Hash. In the experimental settings of reads2graph, a bucket is classified as a large bucket if it contains 10000 or more reads.

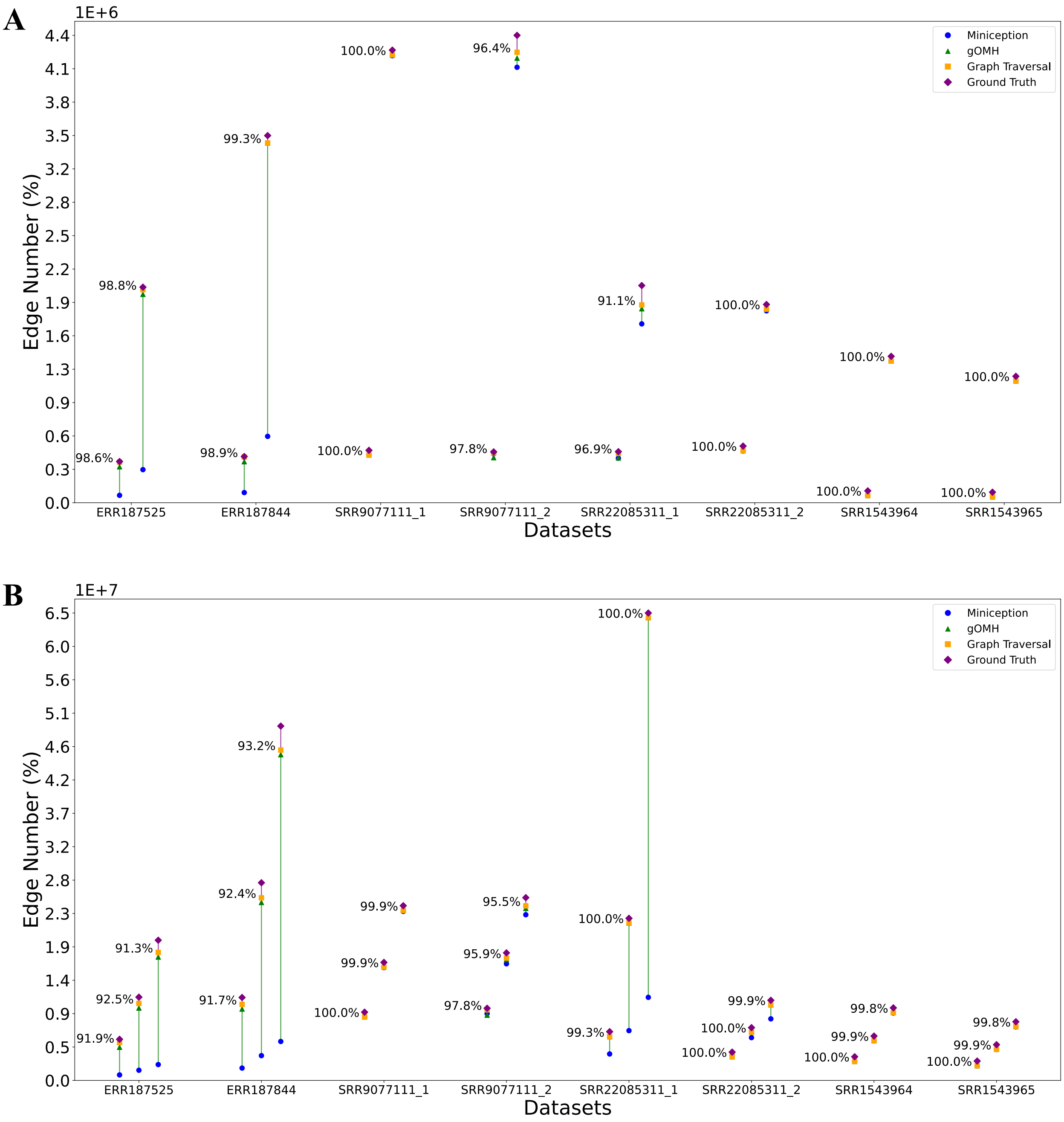

Supplementary Figure 2: Lollipop plot depicting the progression of performance for reads2graph across multiple datasets and intervals, with each marker representing a different stage: Miniception (blue) bucketing without read segmentation, gOMH (green) bucketing, and Graph Traversal (orange). The y-axis shows the number of edges constructed by reads2graph, with the percentage relative to the ground truth labeled next to the final-stage marker. In subplot (**A**), intervals [1,1] (left) and [1,2] (right) are used for each dataset, while subplot (**B**) features intervals [1, 3], [1, 4] and [1, 5] from left to right for each dataset. gOMH: gapped k-mer based Order Min Hash. In the experimental settings of reads2graph, a bucket is classified as a large bucket if it contains 10000 or more reads.

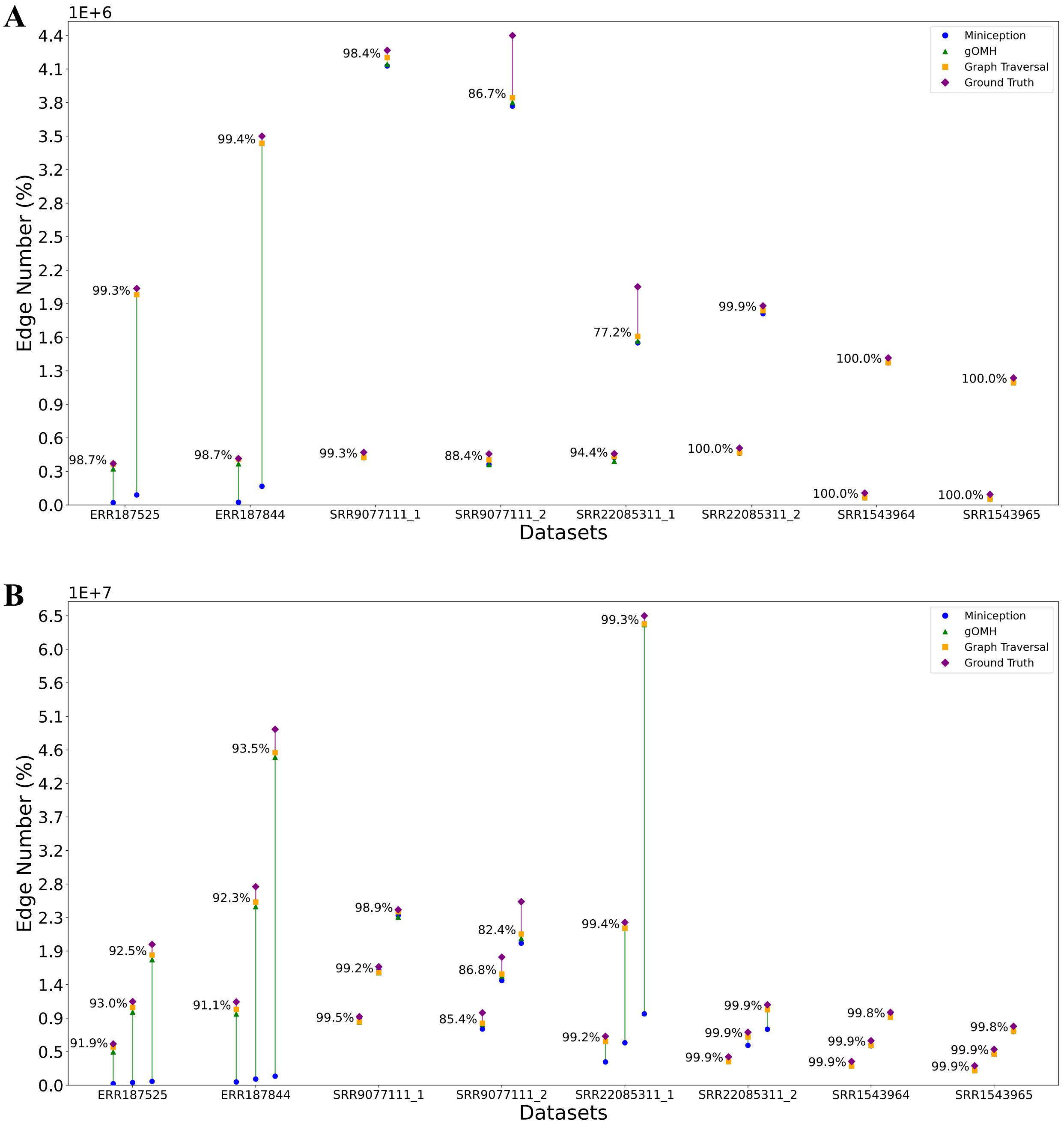

Supplementary Figure 3: Lollipop plot depicting the progression of performance for reads2graph across multiple datasets and intervals, with each marker representing a different stage: Miniception (blue) bucketing with read segmentation, gOMH (green) bucketing, and Graph Traversal (orange). The y-axis shows the number of edges constructed by reads2graph, with the percentage relative to the ground truth labeled next to the final-stage marker. In subplot (**A**), intervals [1,1] (left) and [1,2] (right) are used for each dataset, while subplot (**B**) features intervals [1, 3], [1, 4] and [1, 5] from left to right for each dataset. gOMH: gapped k-mer based Order Min Hash. In the experimental settings of reads2graph, a bucket is classified as a large bucket if it contains 10000 or more reads.

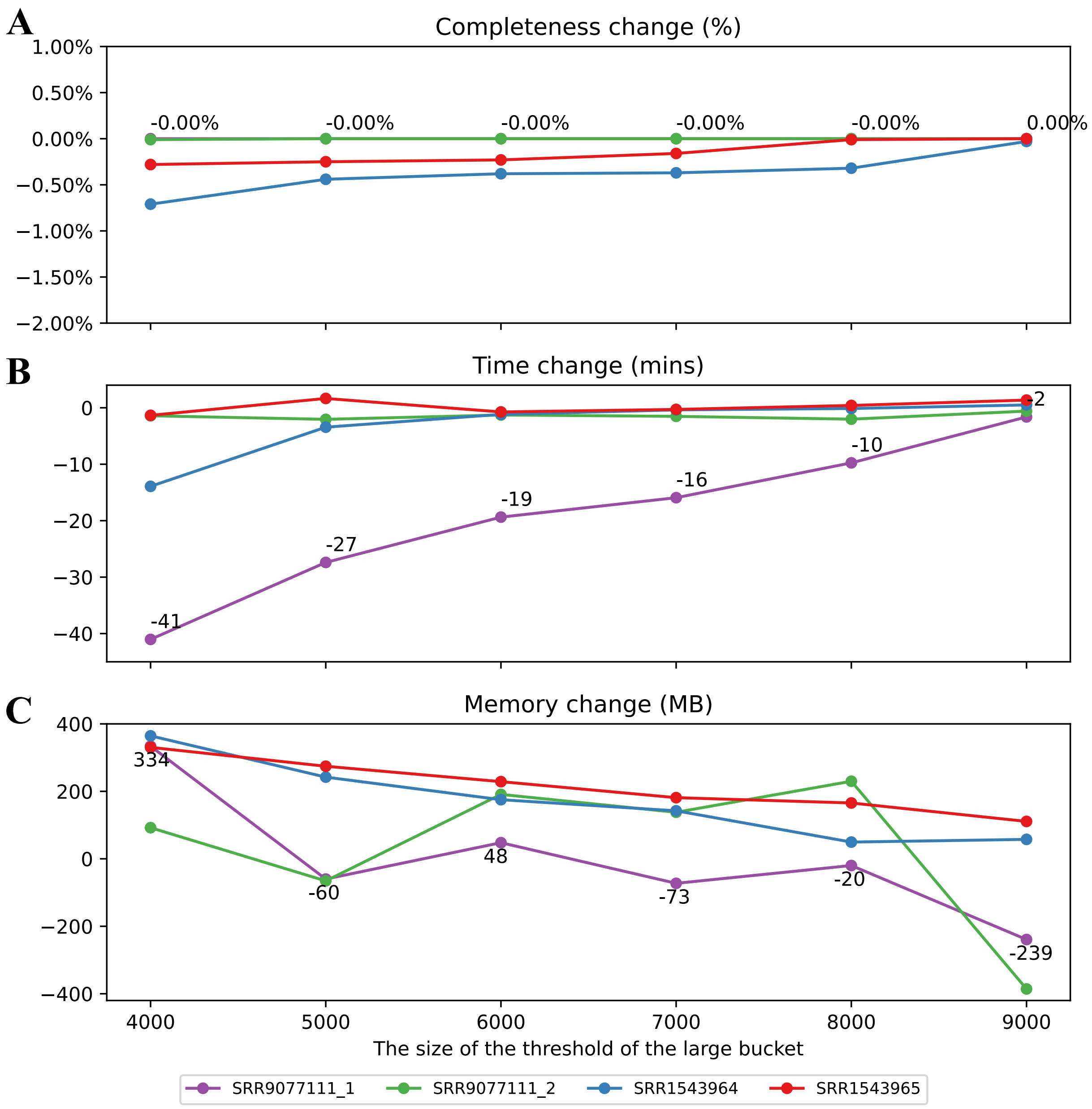

Supplementary Figure 4: Performance of reads2graph using random minimizer bucketing with read segmentation at varying large bucket thresholds. Metrics include changes in completeness (**A**), runtime (**B**), and memory usage (**C**) when constructing read graph with the edit distance interval set to [1, 3].

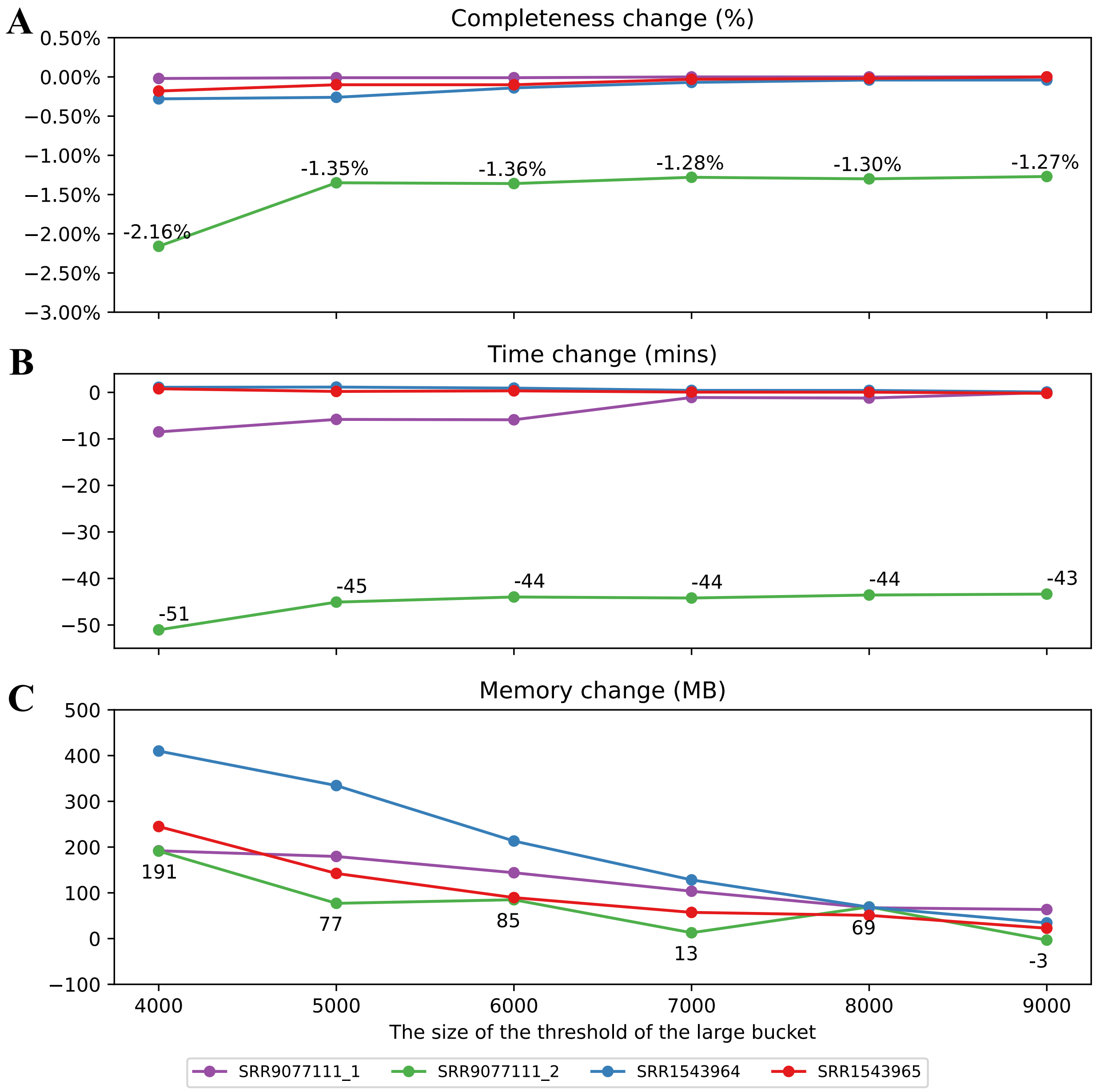

Supplementary Figure 5: Performance of reads2graph using Miniception bucketing without read segmentation at varying large bucket thresholds. Metrics include changes in completeness (**A**), runtime (**B**), and memory usage (**C**) when constructing read graph with the edit distance interval set to [1, 3].
